# Supplementary material for: Sodium Content in Pre-Packaged Foods in China: A Food Label Analysis
Source: Nutrients. 2023 Nov 21;15(23):4862. doi: 10.3390/nu15234862 (PMC10708419; doi:10.3390/nu15234862)
Supplement: Supplementary file 1 [file nutrients-15-04862-s001.zip › Sodium content in pre-packaged foods in China A Food Label Analysis Supplementary Materials.pdf]

Supplementary Materials :

Table S1. Reference standards for the classification of Pre-packaged Foods in China

Food category	Subcategory	Type of standards	Standard
Chocolate, sugar confectionery and Jelly		national standards, health standards	The Standard on Nutrition Labelling of Prepackaged Foods (GB 28050-2011), The Standards for Uses of Food Additive (GB 2760-2014), Regulation of Food Composition Data Expression (WS/T 464-2015), Confectionery (GB17399-2016), Chocolate, cocoa butter substitute chocolate and their products (GB 9678.2-2014), Jelly (GB 19299-2015)
Cake, and pastry products			The Standard on Nutrition Labelling of Prepackaged Foods (GB 28050-2011), The Standards for Uses of Food Additive (GB 2760-2014), Regulation of Food Composition Data Expression (WS/T 464-2015), Pastry, Bread (GB 7099-2015), Moon cake (GB/T 19855-2015)
	Moon cakes	national standards, health standards	The Standard on Nutrition Labelling of Prepackaged Foods (GB 28050-2011), The Standards for Uses of Food Additive (GB 2760-2014), Regulation of Food Composition Data Expression (WS/T 464-2015), Pastry, Bread (GB 7099-2015), Moon cake (GB/T 19855-2015)
	Chinese pastries	national standards, health standards	The Standard on Nutrition Labelling of Prepackaged Foods (GB 28050-2011), The Standards for Uses of Food Additive (GB 2760-2014), Regulation of Food Composition Data Expression (WS/T 464-2015), Pastry, Bread (GB 7099-2015)
	Western-style pastries	national standards, health standards	The Standard on Nutrition Labelling of Prepackaged Foods (GB 28050-2011), The Standards for Uses of Food Additive (GB 2760-2014), Regulation of Food Composition Data Expression (WS/T 464-2015), Pastry, Bread (GB 7099-2015)
Biscuit		national standards, health standards	The Standard on Nutrition Labelling of Prepackaged Foods (GB 28050-2011), The Standards for Uses of Food Additive (GB 2760-2014), Regulation of Food Composition Data Expression (WS/T 464-2015), Biscuit (GB 7100-2015)
	Sandwich biscuits	national standards,	The Standard on Nutrition Labelling of Prepackaged Foods (GB 28050-2011), The

		health standards	Standards for Uses of Food Additive (GB 2760-2014), Regulation of Food Composition Data Expression (WS/T 464-2015), Biscuit (GB 7100-2015)
	Wafer biscuits	national standards, health standards	The Standard on Nutrition Labelling of Prepackaged Foods (GB 28050-2011), The Standards for Uses of Food Additive (GB 2760-2014), Regulation of Food Composition Data Expression (WS/T 464-2015), Biscuit (GB 7100-2015)
	Cookies	national standards, health standards	The Standard on Nutrition Labelling of Prepackaged Foods (GB 28050-2011), The Standards for Uses of Food Additive (GB 2760-2014), Regulation of Food Composition Data Expression (WS/T 464-2015), Biscuit (GB 7100-2015)
	Soda biscuits	national standards, health standards	The Standard on Nutrition Labelling of Prepackaged Foods (GB 28050-2011), The Standards for Uses of Food Additive (GB 2760-2014), Regulation of Food Composition Data Expression (WS/T 464-2015), Biscuit (GB 7100-2015)
	Egg roll	national standards, health standards	The Standard on Nutrition Labelling of Prepackaged Foods (GB 28050-2011), The Standards for Uses of Food Additive (GB 2760-2014), Regulation of Food Composition Data Expression (WS/T 464-2015), Biscuit (GB 7100-2015)
	Other biscuits	national standards, health standards	The Standard on Nutrition Labelling of Prepackaged Foods (GB 28050-2011), The Standards for Uses of Food Additive (GB 2760-2014), Regulation of Food Composition Data Expression (WS/T 464-2015), Biscuit (GB 7100-2015)
Snack foods		national standards, health standards, light industry standards	The Standard on Nutrition Labelling of Prepackaged Foods (GB 28050-2011), The Standards for Uses of Food Additive (GB 2760-2014), Regulation of Food Composition Data Expression (WS/T 464-2015), Extruded snacks (GB 17401-2014), Extruded flavouring noodles (QB/T 5729-2022), General rule for the quality of preserved fruits (GB/T 10782-2021), Non-fried vegetable and fruit crisp chips (GB/T 23787-2009)
	Crisps	national standards, health standards	The Standard on Nutrition Labelling of Prepackaged Foods (GB 28050-2011), The Standards for Uses of Food Additive (GB 2760-2014), Regulation of Food Composition Data Expression (WS/T 464-2015), Extruded snacks (GB 17401-2014)

	Extruded snacks (except for crisps)	national standards, health standards	The Standard on Nutrition Labelling of Prepackaged Foods (GB 28050-2011), The Standards for Uses of Food Additive (GB 2760-2014), Regulation of Food Composition Data Expression (WS/T 464-2015), Extruded snacks (GB 17401-2014)
	Nuts and seeds	national standards, health standards	The Standard on Nutrition Labelling of Prepackaged Foods (GB 28050-2011), The Standards for Uses of Food Additive (GB 2760-2014), Regulation of Food Composition Data Expression (WS/T 464-2015)
	Extruded flavouring noodles	national standards, health standards, light industry standards	The Standard on Nutrition Labelling of Prepackaged Foods (GB 28050-2011), The Standards for Uses of Food Additive (GB 2760-2014), Regulation of Food Composition Data Expression (WS/T 464-2015), Extruded flavouring noodles (QB/T 5729-2022)
	Preserves	national standards, health standards	The Standard on Nutrition Labelling of Prepackaged Foods (GB 28050-2011), The Standards for Uses of Food Additive (GB 2760-2014), Regulation of Food Composition Data Expression (WS/T 464-2015), General rule for the quality of preserved fruits (GB/T 10782-2021)
	Dried fruit	national standards, health standards	The Standard on Nutrition Labelling of Prepackaged Foods (GB 28050-2011), The Standards for Uses of Food Additive (GB 2760-2014), Regulation of Food Composition Data Expression (WS/T 464-2015), Non-fried vegetable and fruit crisp chips (GB/T 23787-2009)
	Soybean curd slab	national standards, health standards	The Standard on Nutrition Labelling of Prepackaged Foods (GB 28050-2011), The Standards for Uses of Food Additive (GB 2760-2014), Regulation of Food Composition Data Expression (WS/T 464-2015)
Beverages		national standards, health standards	The Standard on Nutrition Labelling of Prepackaged Foods (GB 28050-2011), The Standards for Uses of Food Additive (GB 2760-2014), Regulation of Food Composition Data Expression (WS/T 464-2015), Beverage (GB 7101-2022)
Edible ices		national standards, health standards	The Standard on Nutrition Labelling of Prepackaged Foods (GB 28050-2011), The Standards for Uses of Food Additive (GB 2760-2014), Regulation of Food

		Composition Data Expression (WS/T 464-2015), Frozen drinks-Ice cream (GB/T 31114-2014), Frozen drinks-Ice milk (GB/T 31119-2014)
Yogurt, sour milk, cream and similar foods	national standards, health standards,	The Standard on Nutrition Labelling of Prepackaged Foods (GB 28050-2011), The Standards for Uses of Food Additive (GB 2760-2014), Regulation of Food Composition Data Expression (WS/T 464-2015), National food safety standard Pasteurized milk (GB 19645—2010), National food safety standard Sterilized milk (GB 25190—2010), National food safety standard Modified milk (GB 25191—2010), National food safety standard Fermented milk (GB 19302—2010)
Cheese	national standards, health standards	The Standard on Nutrition Labelling of Prepackaged Foods (GB 28050-2011), The Standards for Uses of Food Additive (GB 2760-2014), Regulation of Food Composition Data Expression (WS/T 464-2015)
Convenience foods	national standards, health standards, light industry standards	The Standard on Nutrition Labelling of Prepackaged Foods (GB 28050-2011), The Standards for Uses of Food Additive (GB 2760-2014), Regulation of Food Composition Data Expression (WS/T 464-2015), Instant noodles (GB/T 40772-2021), Instant rice (GB/T 31323-2014), Instant rice noodles (QB/T 2652-2004)
Frozen rice and flour products	national standards, health standards	The Standard on Nutrition Labelling of Prepackaged Foods (GB 28050-2011), The Standards for Uses of Food Additive (GB 2760-2014), Regulation of Food Composition Data Expression (WS/T 464-2015), Quick-frozen dumpling (GB/T 23786-2009)
Cereals	national standards, health standards, local standards	The Standard on Nutrition Labelling of Prepackaged Foods (GB 28050-2011), The Standards for Uses of Food Additive (GB 2760-2014), Regulation of Food Composition Data Expression (WS/T 464-2015), Rolled oats (DB15/T 22952021)
Congee	national standards, health standards	The Standard on Nutrition Labelling of Prepackaged Foods (GB 28050-2011), The Standards for Uses of Food Additive (GB 2760-2014), Regulation of Food Composition Data Expression (WS/T 464-2015), Convenience soup (T/ZSSP 0005-2021)

Edible oil		national standards, health standards	The Standard on Nutrition Labelling of Prepackaged Foods (GB 28050-2011), The Standards for Uses of Food Additive (GB 2760-2014), Regulation of Food Composition Data Expression (WS/T 464-2015), vegetable oil (GB 2716--2018)
Bread and bakery products		national standards, health standards	The Standard on Nutrition Labelling of Prepackaged Foods (GB 28050-2011), The Standards for Uses of Food Additive (GB 2760-2014), Regulation of Food Composition Data Expression (WS/T 464-2015), Pastry, bread (GB 7099-2015)
	Sweet breads	national standards, health standards	The Standard on Nutrition Labelling of Prepackaged Foods (GB 28050-2011), The Standards for Uses of Food Additive (GB 2760-2014), Regulation of Food Composition Data Expression (WS/T 464-2015), Pastry, bread (GB 7099-2015)
	Leavened breads	national standards, health standards	The Standard on Nutrition Labelling of Prepackaged Foods (GB 28050-2011), The Standards for Uses of Food Additive (GB 2760-2014), Regulation of Food Composition Data Expression (WS/T 464-2015), Pastry, bread (GB 7099-2015)
Fine dried noodles		national standards, health standards	The Standard on Nutrition Labelling of Prepackaged Foods (GB 28050-2011), The Standards for Uses of Food Additive (GB 2760-2014), Regulation of Food Composition Data Expression (WS/T 464-2015), Dried noodle (GB/T 40636-2021)
Processed meat products		national standards, health standards, industry standards, group standards	The Standard on Nutrition Labelling of Prepackaged Foods (GB 28050-2011), The Standards for Uses of Food Additive (GB 2760-2014), Regulation of Food Composition Data Expression (WS/T 464-2015), Cured Meat Products (GB 2730-2015), Prefabricated Seasoned Meat Products (T/FJSP 0006-2020), Soy Sauce and Pot-roast Meat Products (GB/T 23586-2009), Dried Meat Floss (GB/T 23968-2009), Dried Meat Dice (GB/T 23969-2009), Dried Meat Slice (GB/T 31406-2015), Bacon (GB/T 23492-2009), Canned Steamed Beef (GB/T 13514-1992), Canned Pork in Natural Juice (GB/T 13513-1992), Canned Steamed Pork (QB/T 2786-2006), Cooked Crued Ham (GB/T 20711-2006), Ham Sausage (GB/T 20712-2006)

Cured meat products	national standards, health standards	The Standard on Nutrition Labelling of Prepackaged Foods (GB 28050-2011), The Standards for Uses of Food Additive (GB 2760-2014), Regulation of Food Composition Data Expression (WS/T 464-2015), Cured Meat Products (GB 2730-2015)
Soy sauce and pot-roast meat products	national standards, health standards,	The Standard on Nutrition Labelling of Prepackaged Foods (GB 28050-2011), The Standards for Uses of Food Additive (GB 2760-2014), Regulation of Food Composition Data Expression (WS/T 464-2015), Soy Sauce and Pot-roast Meat Products (GB/T 23586-2009)
Smoked and roasted meat products	national standards, health standards	The Standard on Nutrition Labelling of Prepackaged Foods (GB 28050-2011), The Standards for Uses of Food Additive (GB 2760-2014), Regulation of Food Composition Data Expression (WS/T 464-2015), Bacon (GB/T 23492-2009)
Sausage meat products	national standards, health standards	The Standard on Nutrition Labelling of Prepackaged Foods (GB 28050-2011), The Standards for Uses of Food Additive (GB 2760-2014), Regulation of Food Composition Data Expression (WS/T 464-2015), Cooked Crued Ham (GB/T 20711-2006), Ham Sausage (GB/T 20712-2006)
Dried meat products	national standards, health standards	The Standard on Nutrition Labelling of Prepackaged Foods (GB 28050-2011), The Standards for Uses of Food Additive (GB 2760-2014), Regulation of Food Composition Data Expression (WS/T 464-2015), Dried Meat Floss (GB/T 23968-2009), Dried Meat Dice (GB/T 23969-2009), Dried Meat Slice (GB/T 31406-2015)
Canned meat	national standards, health standards, industry standards	The Standard on Nutrition Labelling of Prepackaged Foods (GB 28050-2011), The Standards for Uses of Food Additive (GB 2760-2014), Regulation of Food Composition Data Expression (WS/T 464-2015), Canned Stewed Beef (QB/T 1363-1991), Canned Steamed Beef (GB/T 13514-1992), Canned Pork in Natural Juice (GB/T 13513-1992), Canned Steamed Pork (QB/T 2786-2006)
Prepared meat	national standards,	The Standard on Nutrition Labelling of Prepackaged Foods (GB 28050-2011), The

	products	health standards, group standards	Standards for Uses of Food Additive (GB 2760-2014), Regulation of Food Composition Data Expression (WS/T 464-2015), Prefabricated Seasoned Meat Products (T/FJSP 0006-2020)
Processed poultry products		national standards, health standards, group standards	The Standard on Nutrition Labelling of Prepackaged Foods (GB 28050-2011), The Standards for Uses of Food Additive (GB 2760-2014), Regulation of Food Composition Data Expression (WS/T 464-2015), Cured Meat Products (GB 2730-2015), Prefabricated Seasoned Meat Products (T/FJSP 0006-2020), Soy Sauce and Pot-roast Meat Products (GB/T 23586-2009), Dried Meat Floss (GB/T 23968-2009), Dried Meat Dice (GB/T 23969-2009), Dried Meat Slice (GB/T 31406-2015), Bacon (GB/T 23492-2009), Canned Steamed Beef (GB/T 13514-1992), Canned Pork in Natural Juice (GB/T 13513-1992), Canned Steamed Pork (QB/T 2786-2006), Cooked Crued Ham (GB/T 20711-2006), Ham Sausage (GB/T 20712-2006)
	Soy sauce and pot-roast poultry products	national standards, health standards	The Standard on Nutrition Labelling of Prepackaged Foods (GB 28050-2011), The Standards for Uses of Food Additive (GB 2760-2014), Regulation of Food Composition Data Expression (WS/T 464-2015), Soy Sauce and Pot-roast Meat Products (GB/T 23586-2009)
	Smoked and roasted poultry products	national standards, health standards	The Standard on Nutrition Labelling of Prepackaged Foods (GB 28050-2011), The Standards for Uses of Food Additive (GB 2760-2014), Regulation of Food Composition Data Expression (WS/T 464-2015), Bacon (GB/T 23492-2009)
	Sausage poultry products	national standards, health standards	The Standard on Nutrition Labelling of Prepackaged Foods (GB 28050-2011), The Standards for Uses of Food Additive (GB 2760-2014), Regulation of Food Composition Data Expression (WS/T 464-2015), Cooked Crued Ham (GB/T 20711-2006), Ham Sausage (GB/T 20712-2006)
	Prepared poultry products	national standards, health standards, group	The Standard on Nutrition Labelling of Prepackaged Foods (GB 28050-2011), The Standards for Uses of Food Additive (GB 2760-2014), Regulation of Food

		standards	Composition Data Expression (WS/T 464-2015), Prefabricated Seasoned Meat Products (T/FJSP 0006-2020)
Processed fish products		national standards, health standards	The Standard on Nutrition Labelling of Prepackaged Foods (GB 28050-2011), The Standards for Uses of Food Additive (GB 2760-2014), Regulation of Food Composition Data Expression (WS/T 464-2015), National Food Safety Standards of Animal derived aquatic products (GB 10136-2015)
	Cooked fish and seafood products	national standards, health standards	The Standard on Nutrition Labelling of Prepackaged Foods (GB 28050-2011), The Standards for Uses of Food Additive (GB 2760-2014), National Food Safety Standards of Animal derived aquatic products (GB 10136-2015)
	Canned fish	national standards, health standards	The Standard on Nutrition Labelling of Prepackaged Foods (GB 28050-2011), The Standards for Uses of Food Additive (GB 2760-2014), National Food Safety Standards of Animal derived aquatic products (GB 10136-2015)
	Other fish products	national standards, health standards	The Standard on Nutrition Labelling of Prepackaged Foods (GB 28050-2011), The Standards for Uses of Food Additive (GB 2760-2014), National Food Safety Standards of Animal derived aquatic products (GB 10136-2015)
Processed egg products		national standards, health standards	The Standard on Nutrition Labelling of Prepackaged Foods (GB 28050-2011), The Standards for Uses of Food Additive (GB 2760-2014), Regulation of Food Composition Data Expression (WS/T 464-2015), Eggs and egg products (GB 2749-2015)
Sauces, dips and dressings		national standards, health standards, industry standards	The Standard on Nutrition Labelling of Prepackaged Foods (GB 28050-2011), The Standards for Uses of Food Additive (GB 2760-2014), Regulation of Food Composition Data Expression (WS/T 464-2015), Classification of condiment (GB/T 20903-2007), Soy sauces (GB 2717-2018), Pickled vegetables (GB 2714-2015), Ferm ented bean curd (SB/T 10302-1999), Compound seasoning (GB 31644-2018)
	Soy sauces	national standards,	The Standard on Nutrition Labelling of Prepackaged Foods (GB 28050-2011), The

	health standards	Standards for Uses of Food Additive (GB 2760-2014), Regulation of Food Composition Data Expression (WS/T 464-2015), Classification of condiment (GB/T 20903-2007), Soy sauces (GB 2717-2018)
Pickled vegetables	national standards, health standards	The Standard on Nutrition Labelling of Prepackaged Foods (GB 28050-2011), The Standards for Uses of Food Additive (GB 2760-2014), Regulation of Food Composition Data Expression (WS/T 464-2015), Classification of condiment (GB/T 20903-2007), Pickled vegetables (GB 2714-2015)
Fermented bean curd	national standards, health standards, industry standards	The Standard on Nutrition Labelling of Prepackaged Foods (GB 28050-2011), The Standards for Uses of Food Additive (GB 2760-2014), Regulation of Food Composition Data Expression (WS/T 464-2015),Classification of condiment (GB/T 20903-2007), Ferm ented bean curd (SB/T 10302-1999)
Paste and like products	national standards, health standards	The Standard on Nutrition Labelling of Prepackaged Foods (GB 28050-2011), The Standards for Uses of Food Additive (GB 2760-2014), Regulation of Food Composition Data Expression (WS/T 464-2015), Classification of condiment (GB/T 20903-2007)
Compound seasoning	national standards, health standards	The Standard on Nutrition Labelling of Prepackaged Foods (GB 28050-2011), The Standards for Uses of Food Additive (GB 2760-2014), Regulation of Food Composition Data Expression (WS/T 464-2015), Classification of condiment (GB/T 20903-2007), Compound seasoning (GB 31644-2018)
